# Effect of opalescence in resin-based dental composites on the active color matching ability

**DOI:** 10.1186/s12903-024-05233-2

**Published:** 2025-03-18

**Authors:** Joseph Chang, Bum-Soon Lim, Shin Hye Chung, Wonjoon Moon

**Affiliations:** Department of Dental Biomaterials Science and Dental Research Institute, School of Dentistry, Seoul National University, 101 Daehak-Ro, Jongno-Gu, Seoul, 03080 South Korea

**Keywords:** Color matching ability, Opalescence, Resin composite, Color difference, Translucency, Shade

## Abstract

**Objective:**

This study aims to evaluate the influence of opalescence in the color matching ability of dental composites.

**Methods:**

Single-disc samples were prepared using 10 commercially available composites (6 conventional, 3 simplified-shade, and 1 single-shade). Dual-color samples were created using Omnichroma, Ceram.X Sphere TEC (A3 shade), and DenFil (B2/C2 shade), with various cavity diameters in the center, which were then filled with the aforementioned 10 composites. Additionally, single-color samples were prepared using the 4 base composites mentioned above. The ΔE_Single-Dual_ were calculated as differences between single and dual samples. The color coordinates (CIE L^*^a^*^b^*^) of the samples were measured using a spectrophotometer. The results of translucency parameters (TP) and opalescence parameters (OP) of the 10 composites were compared with ΔE measurement samples.

**Results:**

TP and OP generally had a linear correlation, and ΔE_Single-Dual_ mostly increased with cavity diameter. Regarding the correlation between ΔE_Single-Dual_ and TP, little correlation was observed in the conventional composite bases, but higher correlations were observed in the simplified- or single-shade composite bases. Regarding the correlation between ΔE_Single-Dual_ and OP, inverse correlations were observed in the conventional composite bases and simplified composite bases, but the single-shade composite base showed the linear correlations with OP.

**Conclusions:**

These findings highlight a potentially greater role for opalescence in the color matching ability of dental composites than previously acknowledged.

## Background

The esthetic success of dental restorative materials lies in their ability to mimic the optical characteristics of natural teeth. The inherent color of a natural tooth is primarily affected by the shade of the underlying dentin, as well as the thickness, translucency, and light scattering properties of the overlying enamel. Resin-based composites, which are pivotal in dental restorations, have their optical properties shaped by both intrinsic factors—such as the type of matrix and fillers used, and the application technique in the cavity—and extrinsic factors like the illuminant type and light source direction, all of which collectively affect color perception [1–5]. Traditionally, color matching in dental composites has been achieved by incorporating various color pigments, requiring dentists to carefully select composites of different opacities and shades to match the adjacent tooth structures. This process is notably operator-sensitive and time-consuming [6, 7].

In response to these challenges, color adjustment potential (CAP), a material's ability to reduce the perceived color difference compared to viewing those colors in isolation, was introduced to quantify the color matching ability of dental composites [8–10]. In terms of esthetics, the color of the resin-based composite is especially crucial in the success of restoration as it directly interacts with the surrounding color, which determines its CAP [5, 7, 9]. This phenomenon is also referred to as color blending, chameleon effect, or color assimilation [5, 11]. Previously, the color matching ability was mostly investigated through the concept of translucency, which affects light reflectance, diffraction, and the refractive index. Translucency also serves a masking effect, reducing the interaction of light with surrounding structures. Investigating color adjustment through translucency can be considered passive color adjustment and is typically achieved using highly translucent resin-based composites that blend seamlessly with adjacent tooth shades [12–15].

Recently, universal shade composites, with a single-shade or a simplified-shade that is compatible with a wider range of other shades, have been introduced to further advance the color matching ability. The main characteristic of universal resin composites is the reliance on the optical properties of the resin composite to produce color instead of colorants. The first single-shade resin composite introduced to the market, Omnichroma (Tokuyama Dental Corporation, Tokyo, Japan), utilizes structural color as its primary mechanism of color adjustment effect. Researchers have explored the structural color to elucidate the mechanisms behind the derived chameleon effect, where the structure of the composite induces specific light scattering, enhancing or diminishing certain wavelengths. Consequently, this creates a color expression distinct from the material's inherent color, imitating adjacent colors [7, 16–18]. This effect is produced through light reflection, diffuse reflection, diffraction, and interference, facilitated by spatially ordered nano- or microstructures within photonic materials [7, 16, 17]. The inclusion of uniformly distributed zirconium dioxide (ZrO_2_) fillers mixed with supra-nanospherical silicon dioxide (SiO_2_) fillers measuring 260 nm in diameter, alongside round-shaped composite fillers in Omnichroma, results in red-to-yellow structural colors, enhancing color stability by preventing pigment color fading over time [7, 16, 17]. As such, the creation of a resin composite with high color matching ability based on structural color requires a precise amalgamation of optical properties to manipulate light scattering effectively [16, 17].

Opalescence, defined as an optical phenomenon resulting from dispersion of short-wave light within the visible spectrum, causing teeth to appear blue under reflected light and orange under transmitted light [15, 19–21]. This phenomenon is caused by light scattering from particles smaller than the visible light wavelength dispersed through a translucent matrix of a lower refractive index. The opalescence of resin-based composites is usually achieved by the different refractive indices of the resin matrix and the inorganic fillers. The underlying mechanisms dictating structural color may share similarities to those of opalescence in that both are governed by the combinations of light characteristics, such as reflection, refraction, and scattering of incident light [15, 18, 20, 22]. However, past studies have rarely included the evaluation of opalescence in assessing the color matching ability of resin-based composites. Comparative studies investigating the impact of opalescence on the color matching ability are notably less common than those examining translucency. Given that opalescence likely influences the chameleon effect—similarly to how structural color operates through underlying mechanisms—a thorough evaluation of opalescence is essential in studies exploring this effect in resin composites [16].

The purpose of the present study was to evaluate the influence of opalescence in the color matching ability of resin composites. Optical properties and color matching ability were evaluated in single, simplified and conventional nanohybrid resin composites. The null hypothesis for this study is that the opalescence parameter has no effect on the color matching ability of resin composites.

## Methods

### Materials

Ten commercially available resin composites were selected for the evaluation of their color matching ability. These include one single-shade composite (Omnichroma), 3 simplified-shade composites (Ceram.X Sphere TEC, DenFil NX, and Solare Sculpt), and 6 nanohybrid composites (Charisma Diamond, EcuSphere Flow, Estelite S Quick, G-ænial Universal Injectable, Gradia Direct, and Tetric N-Ceram) that are claimed for their color adjustment properties by their manufacturers. The main components, filler fractions, and manufacturer information are listed in Table 1.

**Table 1:** Composite materials used in this study

**Shade**	**Product**	**Resin matrix**	**Filler particle**	**Filler fraction** (wt%)	**Manufacturer**
Single shade					
	Omnichroma (OMC)	UDMA, TEGDMA	Spherical silica-zirconia filler (260 nm) 200–400 nm	79	Tokuyama Dental, Tokyo, Japan
Simplified shade					
A3 for Base	Ceram.X Sphere TEC (CXS)	UDMA, Bis-EMA, TEGDMA	Sphere TEC fillers (15 µm), non-agglomerated Ba-glass (600 nm), YbF_3_ (600 nm)	77–79	Dentsply Sirona, Charlotte, NC, USA
	DenFil NX (DFN)	UDMA, Bis-GMA, TEGDMA, PEGDMA	Silica, spherical filler, Ba-glass	76–78	Vericom, Chuncheon, Korea
	Solare Sculpt (SLS)	Bis-GMA, UDMA, Bis-MEPP, TEGDMA	Ba-glass, fine silica, Sr-glass fillers (300 nm)	73	GC, Tokyo, Japan
Nanohybrid					
	Charisma Diamond (CHD)	TCD-DI-HEA, UDMA, Bis-GMA, TEGDMA	Ba-Al-F glass (5–2,000 nm)	81	Kulzer, Hanau, Germany
	EcuSphere Flow (ESF)	Optimized bis-GMA	Ba-glass	63	DMG, Ridgefield Park, NJ, USA
	Estelite S Quick (ESQ)	Bis-GMA, TEGDMA,	Supra-nano monodispersing spherical filler: SiO_2_-ZrO_2_ average size = 200 nm (100–300 nm)	82	Tokuyama Dental
	G-aenial Universal Injectable (GUI)	UDMA, TEGDMA, Bis-MEPP	Silicon dioxide (16 nm), Sr-glass (200 nm)	69	GC
	Gradia Direct (GRD)	UDMA methacrylate monomers	Microfine silica, pre- polymerized fillers (850 nm)	73	GC
	Tetric N-Ceram (TNC)	Bis-GMA, UDMA, Bis-EMA	Ba-Al–silicate (400–700 nm), YbF_3_ (200 nm), spheroid mixed oxide (120–160 μm)	75–77	Ivoclar Vivadent, Schaan, Liechtenstein
Microhybrid					
B2/C2 for Base	DenFil (DNF)	Bis-GMA, UDMA TEGDMA, PEGDMA	Silica microfillers (0.04 μm), barium aluminosilicate (≤ 1 μm)	76–78	Vericom

### Composite sample preparation for evaluation of Translucency Parameter (TP) and Opalescence Parameter (OP)

**Fig. 1:**
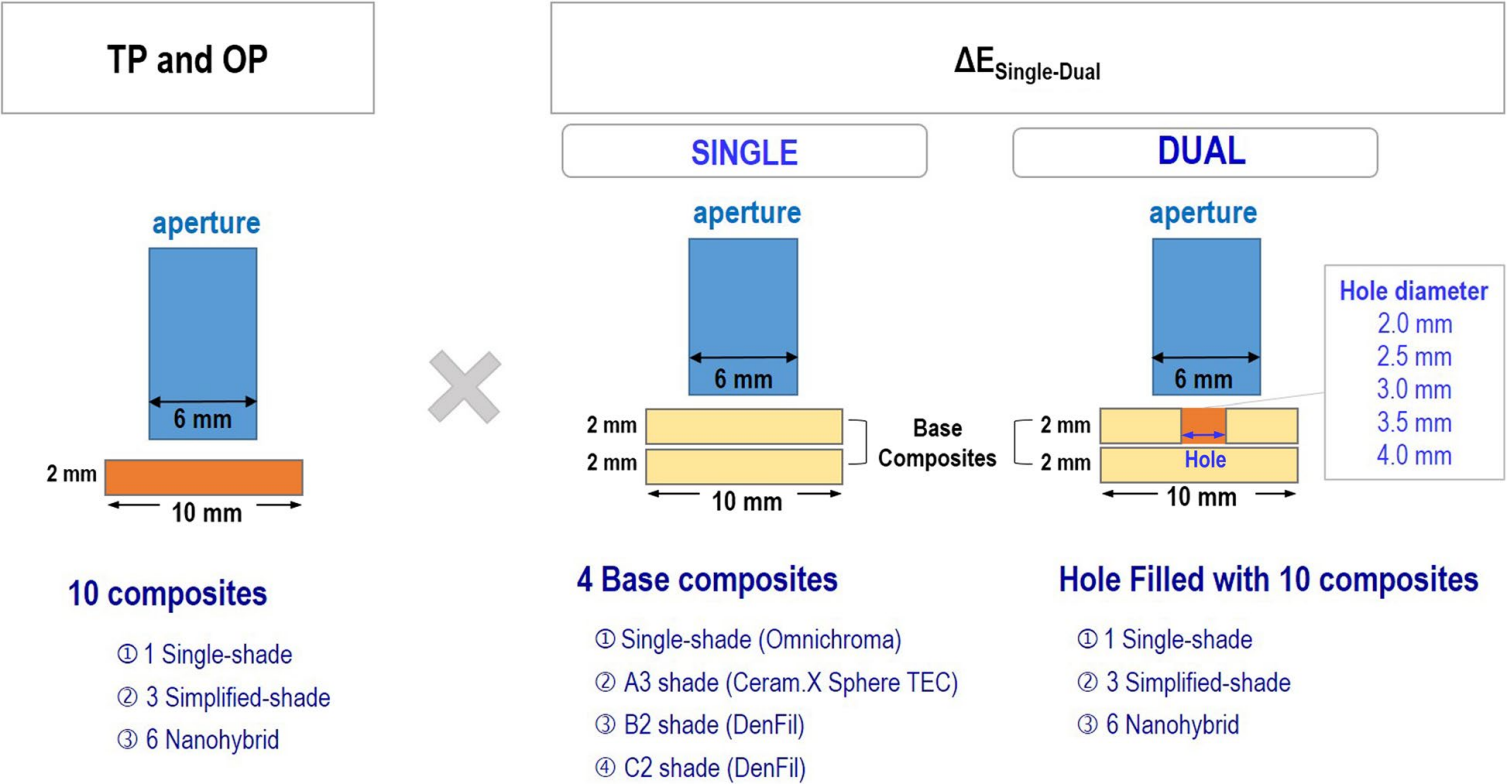
Schematic diagram of sample measurement

Ten resin-based composite discs were prepared for each material (Ø10 mm × 2 mm) (Fig. 1) for the evaluation of TP and OP. Based on a previous study, at least 8 specimens per group were required with 0.05 for the level of significance and 0.46 for the effect size [23]. The sample size was determined as 10 specimens for each group based on the power analysis similar to previous in vitro studies [3, 23–25]. The resin-based composites were applied to a Teflon mold and covered with a mylar strip under a glass slide. The specimens were light-cured at 1400 mW/cm^2^ using Elipar DeepCure-S LED curing light (3 M Oral Care, St. Paul, Minn, USA), for 20 s. The surfaces of all specimens were then polished with SiC paper (#800, #1200, and #2000) under cooling water with a polishing machine, LaboPol-5 (Struers, Copenhagen, Denmark) at 200 rpm. All specimens were then cleaned with sonication in deionized water for 5 min to remove any residual contaminants using a Branson 5510 ultrasonic cleaner (Branson Ultrasonics Co, Danbury, CT, USA) and stored in 37 °C deionized water for 24 h. The thickness of each specimen was measured using a digital micrometer (Mitutoyo Co, Kawasaki, Japan) to ensure consistency. The final thickness was confirmed to be 2.00 mm ± 0.01 mm for all specimens.

### Evaluation of TP and OP

A benchtop sphere spectrometer Ci7600 (X-rite, Grand Rapids, MI, USA) was used to measure the color and optical properties of the specimens. For reflectance measurement, spectral data were recorded from 360 to 750 nm at 10 nm intervals in the reflectance mode with spectral component excluded and UV filter included, against standard white and black backgrounds. The white calibration tile (L = 93.977, a* = 0.017, b* = 2.521) and zero calibration box (L* = 0.033, a* = 0.074, b* = −0.043) provided with the Ci7600 spectrophotometer were used as the backgrounds. Commission Internationale de l’Eclairage (CIE) D65 illuminant with 2° standard observer was used as a light source, and the illuminating and viewing configurations were set at CIE diffuse/8° geometry. The small aperture size (‘SAV’, Ø = 6 mm) and a 6 mm diameter measurement area were used. For transmittance measurement, transmittance mode was used with spectral component included and UV filter included, against black background. CIE D65 illuminant with 2° standard observer was used as a light source, and the illuminating and viewing configurations set at CIE diffuse/0° geometry, with a 6 mm diameter aperture and 6 mm diameter measurement area. The spectral reflectance values were converted into CIELab color coordinates. The L* value represents the brightness of the object, a* value represents the red-green chroma coordinate, and the b* value represents the blue-yellow chroma coordinates [26]. The spectrophotometer was calibrated before the color measurement. Three measurements were taken for each sample and the results for each sample were averaged. TP from the disc (Ø10 mm × 2 mm) samples of 10 composites were measured (Fig. 1) and calculated using the following equation [27–29]:

$$\text{TP}={\left[{\left({L}_{W} {- L}_{B}\right)}^{2}+{\left({a}_{W}-{a}_{B}\right)}^{2}+{\left({b}_{W}-{b}_{B}\right)}^{2}\right]}^{0.5}$$

(where, W = white background, B = black background).

OP from the disc (Ø10 mm × 2 mm) samples of 10 composites were measured (Fig. 1) and calculated using the following equation [22, 31]:

$$\text{OP}={\left[{\left({\text{a}}_{\text{T}}-{\text{a}}_{\text{R}}\right)}^{2}+{\left({b}_{T}-{b}_{R}\right)}^{2}\right]}^{0.5}$$

(where, T = transmittance color, R = reflectance color).

TP and OP of dual samples were also measured to evaluate the correlation with the color differences.

### Composite sample preparation for evaluation of color adjustment effect

Dual composite disc specimens, which consisted of the outer composite (Ø10 mm × 2 mm) with a cavity hole with a diameter of 2.0, 2.5, 3.0, 3.5, and 4.0 mm, filled with other composites, were prepared for the evaluation of the color adjustment effect of resin-based composites. Four resin-based composites were selected to be applied as the outer composites: one single-shade resin composite (Omnichroma), one simplified-shade resin composite (Ceram.X Sphere TEC in A3 shade), and two microhybrid resin composites (DenFil in B2 and C2 shades). The cavity holes were created using a milling machine, Manix MM-180 (Komachine, Yongin, Korea). The specimens were then subjected to sonication in deionized water for 5 min to remove any residual contaminants. The holes were filled with 10 resin composites (Table 1) and covered with a mylar strip under a glass slide then light cured at for 20 s. Samples were finely polished with SiC paper (#800, #1200, and #2000). All samples were cleaned with sonication in deionized water for 5 min. The thickness of all samples was confirmed to be 2.0 ± 0.01 mm using a digital micrometer. For each group, an additional single composite disc specimen of aforementioned four resin-based composites was prepared using the protocol used in the sample preparation for the evaluation of TP and OP to serve as the base composite.

### Evaluation of color difference

For the evaluation of color difference, the dual composite disc specimens were stacked on top of the single base composite disc specimens that matched the outer composites. The color measurement was conducted using the same conditions used for the reflectance measurement for evaluation of TP. The color difference (ΔE_Single-Dual_) was calculated as the differences in the color coordinates between single and dual sample sets against a black background:

$$\eqalign{\Delta {{\rm{E}}_{{\rm{Single - Dual}}}} = {\rm{ [}} & {{\rm{(}}{L_{{\rm{Single}}}} - {L_{Dual}})^2}{\rm{ + (}}{a_{Single}} - {a_{Dual}}{{\rm{)}}^2} \cr & {\rm{ + (}}{b_{Single}} - {b_{Dual}}{{\rm{)}}^2}{{\rm{]}}^{0.5}} \cr}$$

(where, Single = Single composite measurement, Dual = Dual composite measurement).

### Statistical analysis

The statistical differences of TP and OP among the 10 composites were analyzed through ANOVA (*p* < 0.05) in SPSS Statistics 23 (IBM, Armonk, NY, USA). The correlation between OP and TP were analyzed by Pearson correlation and regression analysis (*p* < 0.05). The same correlation analysis was performed between ΔE_Single-Dual_ and TP as well as ΔE_Single-Dual_ and OP.

## Results

### Translucency parameter and opalescence parameter

**Fig. 2:**
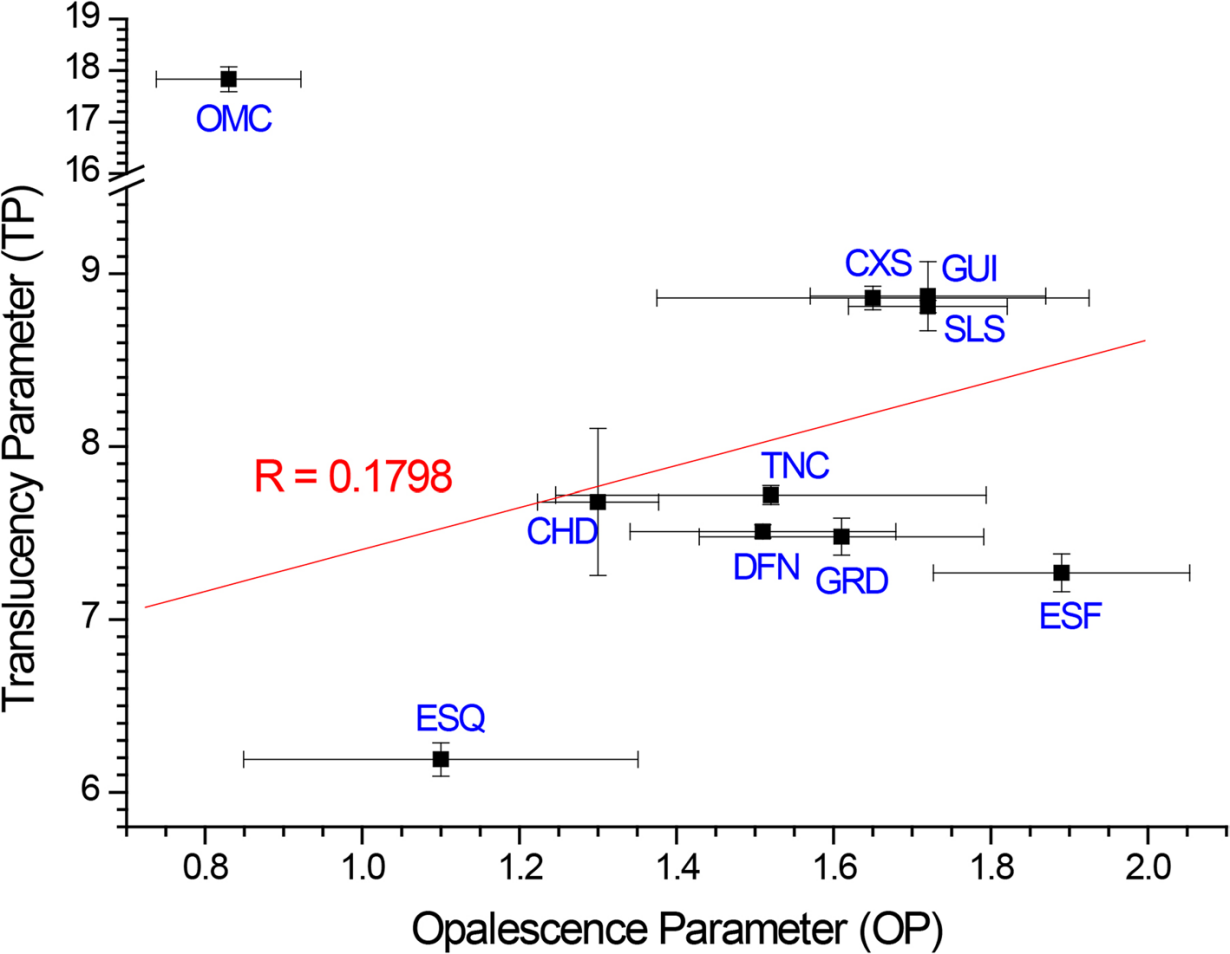
Correlation between OP and TP

TP and OP of the 10 resin composites are summarized in Table 2. Omnichroma showed the highest TP (17.83) and Estelite Sigma Quick showed the lowest TP (6.19). G-aenial Universal Flo showed the highest OP (1.72) and Omnichroma showed the lowest OP (0.83). When the correlation between the opalescence parameter and translucency parameter of resin composites was analyzed, OP generally increased with increasing TP (R = 0.1798) (Fig. 2).

**Table 2:** Translucency parameter (TP) and opalescence parameter (OP) of 10 resin composites

	**Resin composite**	**TP**	**OP**
Single Shade	Omnichroma	17.83 ± 0.24^e^	0.83 ± 0.09^i^
Simplified Shade	Ceram.X Sphere	8.86 ± 0.07^a^	1.65 ± 0.28^f,g^
	Solare Sculpt	8.81 ± 0.04^a^	1.72 ± 0.15^f^
	DenFil NX	7.51 ± 0.04^b,c^	1.51 ± 0.17^f,g^
Nanohybrid	Charisma Diamond	7.68 ± 0.42^b^	1.30 ± 0.08^ g,h^
	EcuSphere Flow	7.27 ± 0.11^c^	1.89 ± 0.16^f^
	Estelite Sigma Quick	6.19 ± 0.10^d^	1.10 ± 0.25^ h,i^
	G-aenial Universal Flo	8.87 ± 0.20^a^	1.72 ± 0.15^f^
	Gradia Direct	7.48 ± 0.11^b,c^	1.61 ± 0.18^f,g^
	Tetric N-Ceram	7.72 ± 0.05^b^	1.52 ± 0.27^f,g^

^*^ Different superscript letters within the column indicate statistically significant difference (*p* < 0.05)

### Color difference (ΔE_Single-Dual_) in relation to cavity diameter

**Fig. 3:**
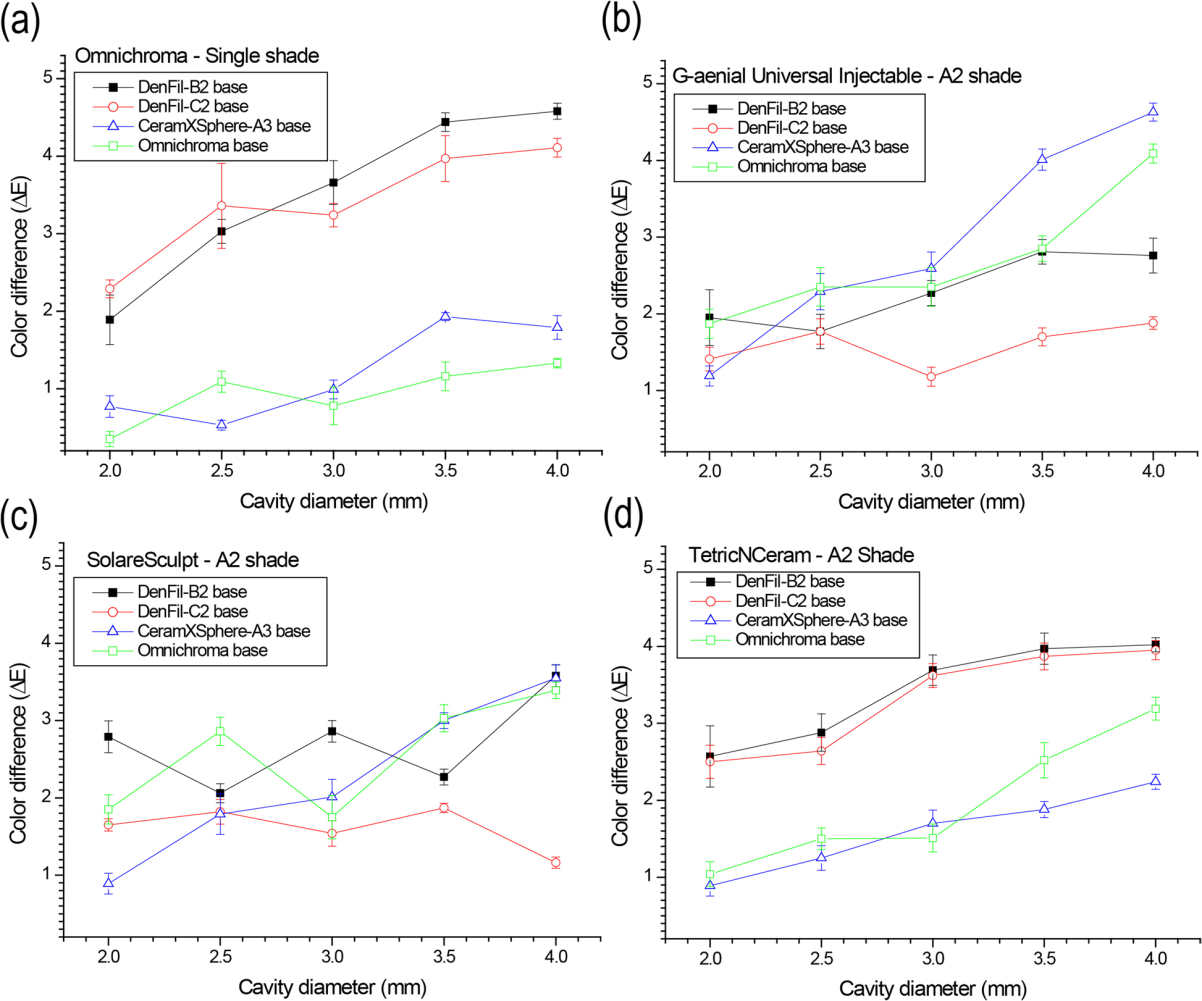
Color difference (ΔE_Single-Dual_) as a function of cavity diameter: (**a**) Omnichroma, (**b**) G-aenial Universal Injectable, (**c**) Solare Sculpt, (**d**) Tetric N-Ceram

The representative results of ΔE_Single-Dual_ as a function of cavity diameter are shown in Fig. 3. In Omnichroma, ΔE tended to increase as the hole size increased (Fig. 3a). Larger color differences were observed in the B2 and C2 microhybrid composite bases than in single-shade and simplified-shade (A2) composite bases. In G-aenial Universal Injectable, ΔE also increased with hole size (Fig. 3b). However, single-shade and simplified-shade bases showed larger ΔE than B2 and C2 bases. Solare Sculpt showed no apparent tendency in relation to cavity diameters and base composites (Fig. 3c). Since the color differences are less than 4 in all groups, acceptable color matching ability can be expected by applying Solare Sculpt. Similar results were observed at Estelite Sigma Quick, Gradia Direct, and Charisma Diamond. In Tetric N-Ceram, larger ΔE was observed in the conventional composite bases than in single-shade and simplified-shade composite bases, and color differences increased as the cavity hole size increased (Fig. 3d). Similar results were observed with Ceram.X Sphere Tec, EcuSphere Flow and DenFil NX.

### Correlation between TP and ΔE_Single-Dual_

**Fig. 4:**
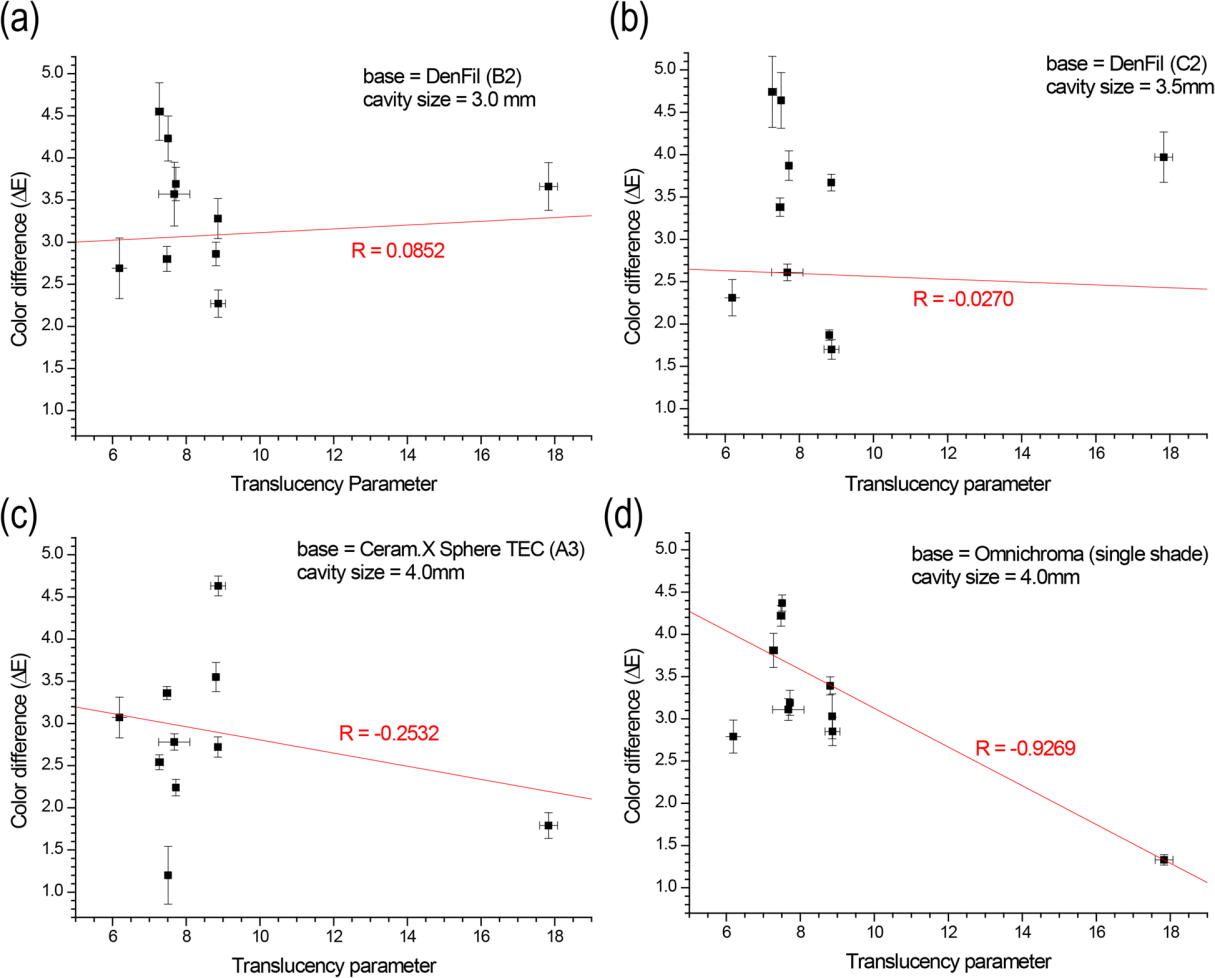
Correlation between color difference (ΔE_Single-Dual_) and translucency parameter (TP) with various resin composite bases and cavity sizes: (**a**) DenFil B2 base, (**b**) DenFil C2 base, (**c**) Ceram.X Sphere TEC (simplified-shade) A3 base, (**d**) Omnichroma (single-shade)

The correlations between TP and ΔE_Single-Dual_ were investigated. Little correlation was observed in the conventional composite bases (*R* = 0.0852, *R* = −0.0270) (Fig. 4a-b); however, higher correlations were observed in the simplified- or single-shade composite bases (*R* = 0.2532, *R* = 0.9269) (Fig. 4c-d). In particular, in the Omnichroma base, ΔE decreased as TP of the cavity-filling composite increased.

### Correlation between OP and ΔE_Single-Dual_

**Fig. 5:**
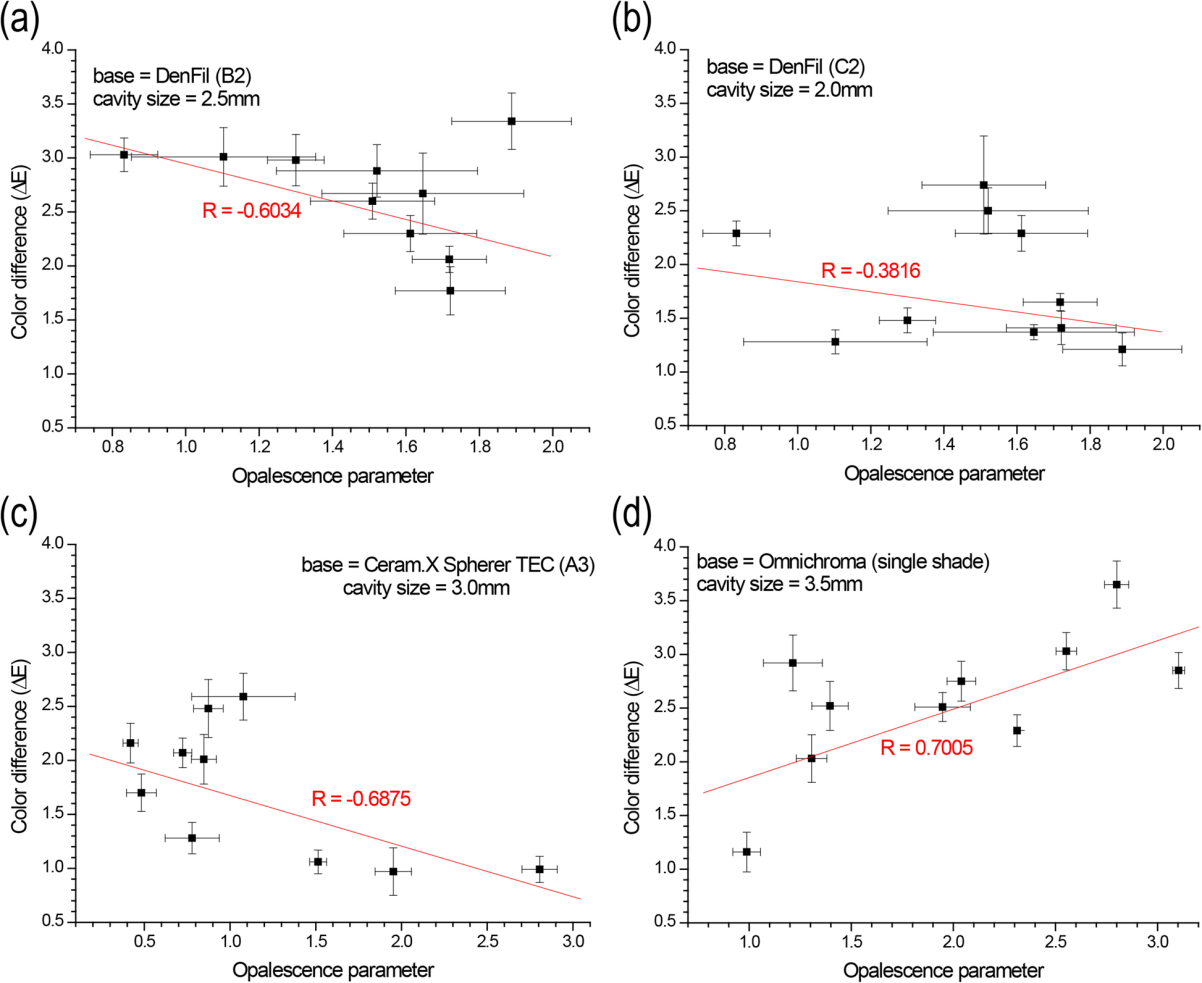
Correlation between color difference (ΔE_Single-Dual_) and opalescence parameter (OP) with various resin composite bases and cavity sizes: (**a**) DenFil B2 base, (**b**) DenFil C2 base, (**c**) Ceram.X Sphere TEC (simplified-shade) A3 base, (**d**) Omnichroma (single-shade)

The correlations between the OP and ΔE_Single-Dual_ were evaluated. Inverse correlations were observed in the B2 and C2 conventional resin composite bases (*R* = −0.6034, *R* = −0.3816) and the A3 simplified resin composite bases (*R* = −0.6875) (Fig. 5a-c). However, the single-shade resin composite base showed linear correlations with OP (*R* = 0.7005) (Fig. 5d). In the Omnichroma base, ΔE increased as the OP of cavity-filling composites increased.

## Discussion

The perceived color of the resin composites is affected by their optical properties. Previously, translucency has been considered integral to determining the color matching ability of the resin composite due to its role in influencing the light transmission characteristics of the material [30]. As a result, recent products such as Omnichroma were designed to have enhanced TP, which was confirmed by the measured values in the current study. This result is also congruent with the findings of previous studies that investigated the optical properties of single-shade resin composites [31, 32]. A recent study compared the TP of various single-shade resin composites and simplified-shade resin composites and observed that Omnichroma exhibited the highest translucency compared to other single-shade resin composites, such as Venus Pearl One and Venus Diamond One (Kulzer GmbH, Hanau, Germany), while the simplified-shade composite exhibited the lowest translucency [31]. The high TP observed for Omnichroma could be attributed to the refractive indices of the filler and the resin matrix of Omnichroma. The translucency of resin composites is highly determined by the refractive index differences between the filler and resin matrix; mismatches in the refractive indices lead to increased light scattering at the resin-filler interface and subsequently increase the opacity of the composite [1, 2, 4, 15, 31, 33]. According to the manufacturer, Omnichroma was fabricated with 79 wt% of uniformly-dispersed 260 nm supra-nanospherical filler so that the refractive index of the resin matrix matches that of the filler [4]. However, in the current study, the previous approach on TP-based color adjustment has been extended to utilizing OP. While translucency is directly influenced by the light transmittance characteristic of the resin composite and is enhanced with greater light transmittance, opalescence benefits from light scattering within the resin matrix. Thus, it has been generally expected for TP and OP to have an inverse relationship. This result has been reported in previous findings that opalescence decreases as translucency increases [12]. However, in the present study, OP exhibited increasing behavior with increasing TP except for Omnichroma (Fig. 2). This implies that increasing OP does not necessarily compromise TP. Rather, TP and OP might coexist to synergistically enhance the color matching ability. Therefore, when Omnichroma exhibited the highest TP but lowest OP, color adjustment by OP could have been underestimated. OP-based color adjustment does not accompany deterioration of TP, which has been known as a critical parameter for CAP. This suggests the novel potential of OP in the color matching ability.

The ΔE increased with the increase in the cavity diameters in the present study (Fig. 3). In the clinical environment, the size of the restoration may vary depending on the size of the tooth cavity; thus, this part of the study was performed to better understand the effects of restoration size on the color matching ability. To our knowledge, no previous study has investigated the effect of restoration size on the color matching ability of resin composites surrounded by different resin composites. The results of the present study indicate that the color matching ability is lower when the restoration size increases. This may arise mainly from the changes in the light transmittance characteristics; as the restoration size increases, the amount of light transmitted to the matrix increases and negatively affects the perceived color. Furthermore, the increased surface area of resin composites that were filled in the cavity hole would be expected to yield a higher color difference as the proportion of restoration to base resin composites would increase.

In the present study, both the TP and OP were found to be correlated with the ΔE. Therefore, the null hypothesis that the opalescence parameter would have no effect on the color matching ability of different types of resin composites was rejected. Regarding translucency, the correlations between the TP and ΔE for the single-shade and simplified-shade composite bases were observed to be higher compared to those of conventional resin composites, with the strongest correlation observed for Omnichroma bases (*R* = 0.9269) (Fig. 4). This result indicates that translucency is more influential for the color matching abilities of single-shade and simplified-shade resin composites. As translucency plays a critical role in determining the light transmittance characteristics of the material, single-shade resin composites rely heavily on translucency to obtain the desired shade; however, conventional resin composites utilize colorants to manually achieve the desired shade and light scattering. Therefore, a lower impact of translucency on the color matching ability of conventional resin composites could be expected. Multiple studies investigating the role of translucency in resin composites have previously reported on the strong correlation between TP and the CAP [27, 29, 34]. The result of the present study also confirms the strong correlation between the TP and the color matching ability.

Regarding opalescence, a linear correlation between the OP and ΔE was observed for the single-shade composite bases, indicating that the color matching ability decreased as opalescence increased (Fig. 5d). Several studies in the past have reported on the effect of light properties on the translucency and opalescence of restorative materials [35–37]. Since both the translucency and opalescence are sensitive to the changes in light transmittance characteristics which could alter properties, such as light reflection and scattering, it can be expected that the color matching ability would decrease with the increase in opalescence. The increase in opalescence often indicates an increase in light scattering, which impairs translucency of the material, which is closely related to the color matching ability. For conventional and simplified-shade composite bases, however, the opalescence parameter and ΔE were found to be inversely correlated, indicating greater color matching ability for conventional and simplified-shade composites under increasing opalescence. Unlike single-shade resin composite, simplified-shade and conventional composites utilize colorants to obtain the desired shades and require expertise of clinicians to manually compare the shades to achieve esthetic success. Consequently, the optical properties required for conventional and simplified-shade resin-based composites would be different from that of single-shade composites.

The interplay between opalescence and translucency in resin composites can be complex. While increased opalescence typically enhances the scattering of light, potentially decreasing overall translucency, it does not necessarily diminish the material’s color matching ability. In fact, as indicated in the current study, the presence of opalescence might enhance color matching ability by providing a more nuanced manipulation of light within the composite, allowing for a more precise color matching that accounts for the depth and subtlety of natural tooth color variations. This suggests that optimizing opalescence could lead to an improvement in how composites perform esthetically, particularly in challenging clinical scenarios where precise color matching is crucial. A notable strength of the present study included that it measured the color of both the resin composite and the surrounding outer composite layer, a methodological decision that more closely simulates the conditions encountered in a clinical setting. This approach allows for a more realistic representation of how the composite interacts with natural tooth structure and external factors, such as light exposure, which can significantly influence the perceived color match. However, a limitation of the study is the absence of a visual assessment of color matching. While instrumental measurements provide valuable insights into the optical properties of the materials, it has been suggested that both the instrumental and visual assessment should be considered, as the chameleon effect could ultimately be described as an optical illusion [5].

Given these considerations, future research should focus on quantifying the specific contributions of opalescence to the color matching ability and exploring how these effects can be manipulated through material science, along with the visual assessment. Investigating the specific mechanisms by which opalescent particles scatter light could lead to the formulation of composites that better control the diffusion of light, thereby enhancing the chameleon effect and improving the visual integration of the restoration with the natural dentition. This could involve experimenting with different sizes, shapes, and concentrations of opalescent fillers to optimize their light-scattering properties. Additionally, the study of opalescence should also consider its impact on the long-term durability and visual stability of composites under various oral conditions, such as exposure to different types of lighting and the effects of aging or staining substances. Understanding these factors will enable the development of resin composites that maintain their esthetic properties over time, thereby improving patient satisfaction and clinical outcomes.

## Conclusions

Within the limitations of the present study, the results demonstrated that the translucency and opalescence parameters significantly affect the color adjustment potentials of various types of resin composites. The present study could highlight the role of opalescence in the color adjustment potential of resin composites as being greater than previously acknowledged. While the appropriate level of translucency is critical in obtaining the optimal color adjustment potential of resin composites, the precise level of opalescence may also be equally important for achieving the optimal color adjustment effect. Future studies should focus more on the role of opalescence in the color adjustment effect of resin composites and investigate the interplay between translucency and opalescence to better understand the color adjustment effects of resin composites.

## Data Availability

The datasets used and/or analyzed during the current study are available from the corresponding author on reasonable request.

## Abbreviations

CAP: Color adjustment potential
TP: Translucency parameter
OP: Opalescence parameter
